# The protease inhibitor gabexate mesylate targets Raf kinase inhibitor protein and reverses epithelial–mesenchymal transition in triple-negative breast cancer cells

**DOI:** 10.3389/fonc.2026.1713273

**Published:** 2026-03-02

**Authors:** Marzia Deserti, Simone Sabbioni, Francesca Poli, Francesco Vasuri, Valeria Relli, Andrea Palloni, Chiara Deiana, Giovanni Brandi, Simona Tavolari

**Affiliations:** 1 Department of Medical and Surgical Sciences, University of Bologna, Bologna, Italy; 2 Pathology Unit, Azienda Unità Sanitaria Locale (AUSL) of Imola, Imola, Italy; 3 Pathology Unit, Santa Maria delle Croci Ravenna Hospital, Ravenna, Italy; 4 Medical Oncology, IRCCS Azienda Ospedaliero-Universitaria di Bologna, Bologna, Italy

**Keywords:** EMT, gabexate mesylate, RKIP, TNBC, tumor invasion

## Abstract

The prognosis of triple-negative breast cancer (TNBC) still remains poor, mainly due to the occurrence of early metastases and the lack of effective treatments. The Raf kinase inhibitor protein (RKIP) is a tumor metastasis suppressor frequently downregulated in human cancers. Here, we report that RKIP expression is lost in the tumor tissue of TNBC patients. Treatment with the protease inhibitor gabexate mesylate (GM) increased RKIP expression and reverted the epithelial–mesenchymal transition (EMT) phenotype in MDA-MB-231 cells, a well-established *in vitro* TNBC model. This phenotypic change was concomitant with the upregulation of the epithelial markers E-Cadherin and Claudin-1, and the downregulation of the mesenchymal markers N-Cadherin, Vimentin, and nuclear β-catenin. Furthermore, GM significantly decreased TNBC cell motility and invasiveness, along with matrix metalloproteinase (MMP)-2 and MMP-9 protein expression and activity. At the mechanistic level, RKIP upregulation inhibited p42/44 mitogen-activated protein kinase (MAPK) and NF-κB signaling that, in turn, downregulated the expression of the two EMT transcription factors Snail and Slug. Despite these preliminary findings needing to be confirmed in more representative *ex vivo* (such as patient-derived primary cells or patient-derived organoids) and *in vivo* TNBC models, they provide evidence that the EMT process could be pharmacologically reverted by the protease inhibitor GM in a highly metastatic TNBC *in vitro* model, deserving further investigation in future studies.

## Introduction

Triple-negative breast cancer (TNBC) accounts for up to 15% of all diagnosed breast cancers (BCs) (1) and encompasses multiple entities defined by the lack of estrogen and progesterone receptors, and human epidermal growth factor receptor 2 (HER2) amplification and/or overexpression. Compared to other BC subtypes, TNBC is associated with the worst prognosis, with a 5-year survival rate ranging from 4% to 20% (2). The higher prevalence in younger women, the intrinsic aggressiveness, and the high relapse rate make TNBC one of the most challenging tumors to be tackled. Metastatic spread usually occurs early in the natural history of this disease, often within the first year after diagnosis, representing the major cause of morbidity and mortality in these patients (3). In this scenario, preventing metastatic progression remains one of the most promising therapeutic strategies against TNBC.

The epithelial–mesenchymal transition (EMT) is a crucial event driving tumor metastasis. Recent findings have emphasized the central role of this process in TNBC aggressiveness and resistance to conventional therapies (4, 5). During the EMT program, cancer cells undergo substantial phenotypic changes, progressively losing epithelial morphology and acquiring a mesenchymal, fibroblast-like phenotype, along with enhanced invasive behavior. These phenotypic changes are typically accompanied by some molecular changes, including the downregulation of epithelial cell adhesion markers and the upregulation of mesenchymal markers (6).

The Raf kinase inhibitor protein (RKIP) has emerged as a metastasis suppressor gene frequently downregulated in human cancers and further reduced in distant metastases (7). Several studies have shown that RKIP expression counteracts the metastatic process at multiple levels, mostly by inhibiting different oncogenic pathways (8), including the mitogen-activated protein kinase (MAPK) and nuclear factor kappa-light-chain-enhancer of activated B cells (NF-κB). Furthermore, RKIP induction has been shown to inhibit the EMT process (9). Loss of RKIP expression represents an important indicator of poor prognosis in several types of malignancies, and its restoration has been reported to inhibit metastasis in different cancer models, including BC (10–13). Accordingly, RKIP has become an attractive therapeutic target for the treatment of highly invasive and metastatic cancers as TNBC.

Interestingly, we previously reported that the protease inhibitor gabexate mesylate (GM) can induce RKIP expression in pancreatic cancer cells at a dose achievable in human blood (14). In light of the growing evidence showing a role for RKIP as a metastasis suppressor gene, here, we investigated the effects of GM treatment in modulating RKIP expression and the invasive phenotype of TNBC cells. The molecular mechanisms on the basis of these effects were also investigated.

## Materials and methods

### Patients

A total of 10 TNBC patients who underwent quadrantectomy or mastectomy at S. Maria della Scaletta Hospital in Imola (Italy) from 2009 to 2014 were retrospectively evaluated. None of them received chemotherapy and radiotherapy before surgery. Tumor clinicopathological features were collected from medical records and matched with pathological evaluation on hematoxylin and eosin-stained slides. All patients included in this study were women aged from 33 to 79 years. The tumor size, nodal involvement, and surgical margins were reported. Tumor samples were histologically classified and graded according to WHO guidelines and pTNM [American Joint Committee on Cancer (AJCC)] pathological staging criteria (15). Hormone receptor expression and HER2 status were assessed via immunohistochemical analysis according to the American Society of Clinical Oncology/College of American Pathologists guidelines (16, 17).

According to the general authorization to process personal data for scientific research purposes from “The Italian Data Protection Authority” (https://www.garanteprivacy.it/home), no ethical approval was required for the study. This study was carried out in accordance with the ethical guidelines of the Declaration of Helsinki (and subsequent modifications).

### Immunohistochemistry

Formalin-fixed, paraffin-embedded (FFPE) TNBC and matched normal mammary tissue samples were collected from patients. Immunohistochemistry (IHC) was carried out using Novolink Polymer Detection System (Leica Microsystems, Wetzlar, Germany), as previously reported (18). Sections were incubated overnight at 4 °C with RKIP (clone D42F3, Cell Signaling, Danvers, MA, USA), E-Cadherin (clone 24E10, Cell Signaling, Danvers, MA, USA), and Vimentin (clone V9; Dako, Hamburg, Germany) primary antibodies. Sections were developed in 3,3′-diaminobenzidine and nuclei counterstained with hematoxylin. RKIP and Vimentin immunostaining was scored according to the following arbitrarily assigned semi-quantitative criterion: (0), 0% of immunoreactive cells; (1), <5% of immunoreactive cells; (2), 5%–50% of immunoreactive cells; and (3), >50% of immunoreactive cells. Samples with scores 0 and 1 were considered negative, and those with scores 2 and 3 were positive. For E-Cadherin, positivity on the cell membrane was assessed as present (≥10% of positive tumor cells) or absent, as previously reported (19). All slides were evaluated by an expert pathologist blinded to the patient’s clinical features.

### Cell culture

MDA-MB-231 cells were obtained from American Type Culture Collection (ATCC; Manassas, VA, USA), cultured in Dulbecco’s modified Eagle’s medium with 4.5 g/L glucose (Euroclone, Milan, Italy), and supplemented with 10% (v/v) heat-inactivated Fetal bovin serum (FBS) (Euroclone), 2 mM l-glutamine, 100 U/mL penicillin, and 100 μg/mL streptomycin (Sigma-Aldrich, St. Louis, MO, USA). Cells were grown at 37 °C in a humidified atmosphere enriched with 5% CO_2_ and routinely passaged using trypsin-EDTA 0.025% (Sigma-Aldrich).

### Drug treatment

GM (Foy, Sanofi Aventis, Milan, Italy) was freshly diluted in serum-free medium to obtain the final concentration of 50 μg/mL before each experiment. This dose was chosen according to the maximal concentration detected in the plasma of healthy volunteers, as previously reported (14).

### Cell viability assay

Cell viability was assessed using 3-(4,5-dimethylthiazol-2-yl)-2,5-diphenyltetrazolium bromide (MTT) assay, as previously reported (14). Briefly, MDA-MB-231 cells (3 × 10^3^ cells/well) were plated in a 96-well plate in triplicate and allowed to adhere for 24 hrs. Cells were then treated with GM 50 μg/mL for 24, 48, and 72 hrs. At the end of incubation, MTT was added to each well, and cells were incubated at 37 °C for 4 hrs. Formazan crystals were then dissolved by Dimethyl sulfoxide (DMSO) addition. Absorbance was then measured at 570 nm in a 96-well spectrophotometric microplate reader (Bio-Rad, Hercules, CA, USA).

### Wound-healing scratch assay

Real-time analysis of cell migration was performed using the IncuCyte^®^ S3 Live-Cell Analysis System (Sartorius, Ann Arbor, MI, USA) according to the manufacturer’s instructions. Briefly, 2 × 10^4^ MDA-MB-231 cells/well were seeded into ImageLock™ 96-well plates and cultured for 24 hrs at 37 °C in a humidified atmosphere (5% CO_2_). Wounds were created in all wells using the WoundMaker™. After gently washing the wells twice with phosphate-buffered saline (PBS), 100 μL of medium containing 50 μg/mL GM was applied to each well. Cell migration was monitored by phase-contrast imaging using an IncuCyte ZOOM^®^ microscope, acquiring an image every 3 hrs for 4 days during constant incubation at 37°C in a humidified atmosphere with 5% CO_2_. The IncuCyte ZOOM^®^ image analysis software was used to detect cell edges automatically and to generate an overlay mask for wound width calculation.

### Invasion assay

The invasive potential of MDA-MB-231 cells was assessed using the Boyden chamber invasion assay. Briefly, 12-μm polyvinylpyrrolidone-free polycarbonate filters (Millipore Co., Cork, Ireland) were coated with growth factor-reduced Matrigel (BD Biosciences, Milan, Italy). Complete medium was placed in the lower chamber as chemo-attractant. MDA-MB-231 cells (7.5 × 10^4^), previously treated with GM 50 μg/mL for 72 hrs, were seeded in the upper chamber and incubated for 6 hrs at 37°C in a humidified atmosphere with 5% CO_2_. At the end of incubation, non-invading cells were removed from the upper surface of the filters, and invading cells on the lower surface were fixed for 1 min in ethanol 95% and stained for 10 min with 0.5% w/v toluidine blue. For each sample, four random optical fields at ×200 total magnification were analyzed, and the mean number of invading cells was calculated.

### Western blotting

Total protein extracts were collected using the ReadyPrep Protein Extraction Kit (Bio-Rad) following the manufacturer’s protocol. Equal amounts (50 µg) of protein extracts were resolved with a 4%–12% gradient Sodium dodecyl sulfate–polyacrylamide gel electrophoresis (SDS–PAGE) and then transferred onto a nitrocellulose membrane with 0.45-mm pore size (Amersham Biosciences, Buckinghamshire, UK). Membranes were incubated overnight at 4 °C with the following primary antibodies, all from Cell Signaling Technology (Danvers, MA, USA): rabbit anti-RKIP (#4742), rabbit anti-Vimentin clone D21H3 (#5741), rabbit anti-N-Cadherin (#13116), rabbit anti-Claudin-1 (#13255), rabbit anti-Snail (#3879), rabbit anti-Slug (#9585), rabbit anti-E-Cadherin (#3195), rabbit anti β-Catenin clone D10A8 (#8480), rabbit anti-MMP-2 (#4022), rabbit anti-MMP-9 (#3852), rabbit anti-phosphorylated IkBα Ser32 clone 14D4 (#2859), mouse anti-IkBα clone L35A5 (#4814), rabbit anti-RelA/p65 clone C22B4 (#4764), rabbit anti-phospho-MEK1/2 Ser217/221 (#9121), rabbit anti-MEK1/2 (#9122), mouse anti-phospho-p44/42 Thr202/Tyr204 (#9101), rabbit anti-p44/42 (#9102), and rabbit anti-β-actin (#4967). Goat anti-rabbit and anti-mouse horseradish peroxidase-conjugated secondary antibodies were from Amersham Biosciences (Little Chalfont, UK). Membranes were stripped using Re-Blot Plus Western Blot Recycling Kit (Chemicon International, Temecula, CA, USA), incubated with specific antibodies, and finally developed using chemiluminescence ECL reagent (Amersham Biosciences). ChemiDoc XRS + (Image Lab Software, Bio-Rad) was employed to acquire and quantify digital images.

### Gelatin zymography

The activity of matrix metalloproteinases 2 and 9 (MMP-2 and MMP-9) was assessed via gelatin zymography. Briefly, MDA-MB-231 cells at approximately 80% of confluence were treated with GM 50 μg/mL for 72 hrs. At the end of incubation, the conditioned medium was collected, and MMP-2/MMP-9 protein activity was assessed as previously reported (14).

### Immunofluorescence and confocal microscopy

MDA-MB-231 cells were plated on glass coverslips and fixed with 4% paraformaldehyde in PBS for 20 min. Permeabilization and blocking were performed in PBS with 10% FBS and 0.1% saponin. Primary rabbit anti-E-Cadherin (#ab40772) and rabbit anti-β-Catenin (#ab6302) antibodies were from Abcam (Cambridge, UK), and Alexa 488-conjugated anti-rabbit IgG (#A11008) and anti-mouse IgG (#A27023) were from Invitrogen (Waltham, MA, USA). Coverslips were mounted using 0.5% *p*-phenylenediamine in 20 mM Tris 8.8 and 90% glycerol. Images were acquired using a confocal Nikon Eclipse T12 microscope (Nikon Instruments Italia, Florence, Italy).

### Statistical analysis

Data were analyzed using the GraphPad Prism software Ver 5.01 (GraphPad Software, San Diego, CA, USA). The Shapiro–Wilk test was used to confirm the Gaussian distributions of raw data. The non-parametric Mann–Whitney U-test was used for comparison of IHC scores. For normally distributed data, differences between groups were analyzed using an unpaired Student’s t-test, and reproducibility was confirmed in three independent experiments. A p-value < 0.05 was considered statistically significant.

## Results

### RKIP protein expression is lost in TNBC tissue

Ten TNBC cases that had undergone quadrantectomy or mastectomy from 2009 to 2014 were retrospectively evaluated. Baseline characteristics of patients are shown in **Table 1**. All patients underwent surgical treatment, with resection margins negative for tumor infiltration. In line with literature data, 70% of patients were younger than 60 years, and 90% were high-grade tumors (G3). Although invasive ductal carcinoma was the most prevalent histological subtype (9/10), one case of medullary-type carcinoma was also included. Of the cases, 80% were early-stage breast cancers (Stage I–IIb).

**Table 1:** Baseline characteristics of the 10 TNBC patients included in the study.

Patient ID	1	2	3	4	5	6	7	8	9	10
Age	59	34	71	72	48	79	58	52	33	47
Date	7/4/2009	07/21/2009	10/23/2009	08/18/2010	09/28/2010	7/4/2011	1/3/2012	2/12/2013	1/2/2014	6/7/2014
Histological subtype	IDC	IDC	IDC	IDC	IDC	IDC	IDC	IDC	IDC	IDC
Histological grade	3	3	3	2	3	3	3	3	3	3
Estrogen receptor (ER) status	Neg	Neg	Neg	<10%	Neg	Neg	Neg	Neg	<1%	Neg
Progesteron receptor (PR) status	Neg	Neg	Neg	<10%	Neg	Neg	Neg	Neg	Neg	Neg
Her-2	0	0	0	0	0	0	0	0	0	0
Ki-67	55%	45%	70%	14%	40%	40%	70%	95%	80%	70%
Size (cm)	1.5	3,5	3,5	1	>5	1.7	3.7	1,7	2	0,9
pTNM	T1cN0	T2N1a	T2N0	T1bN0	T3N3a	T1cN0(i+ sn)	T2N0(sn)	T1cN0(sn)	T1cN1a	T1bN0(sn)
Stage (AJCC 2017 - asg)	Ia	IIb	IIa	Ia	IIIc	Ia	IIa	Ia	IIa	Ia
Stage (AJCC 2017 - ppsg)	Ib	IIIa	IIa	Ia	IIIc	Ib	IIa	Ib	IIa	Ib
Surgical margins	Neg	Neg	Neg	Neg	Neg	Neg	Neg	Neg	Neg	Neg
Vascular invasion (Y/N)	n.a.	Y	Y	n.a.	Y	n.a.	n.a.	Y	Y	N
Perineural invasion (Y/N)	n.a.	n.a.	Y	n.a.	n.a.	n.a.	n.a.	n.a.	n.a	n.a.

IDC=invasive ductal carcinoma; n.a.=not available; Y/N=Yes/Not; Neg=Negative; AJCC asg=American Joint Commitee on Cancer anatomic staging groupings; AJCC ppsg=American Joint Commitee on Cancer pathologic prognostic staging groupings

**Figure 1:**
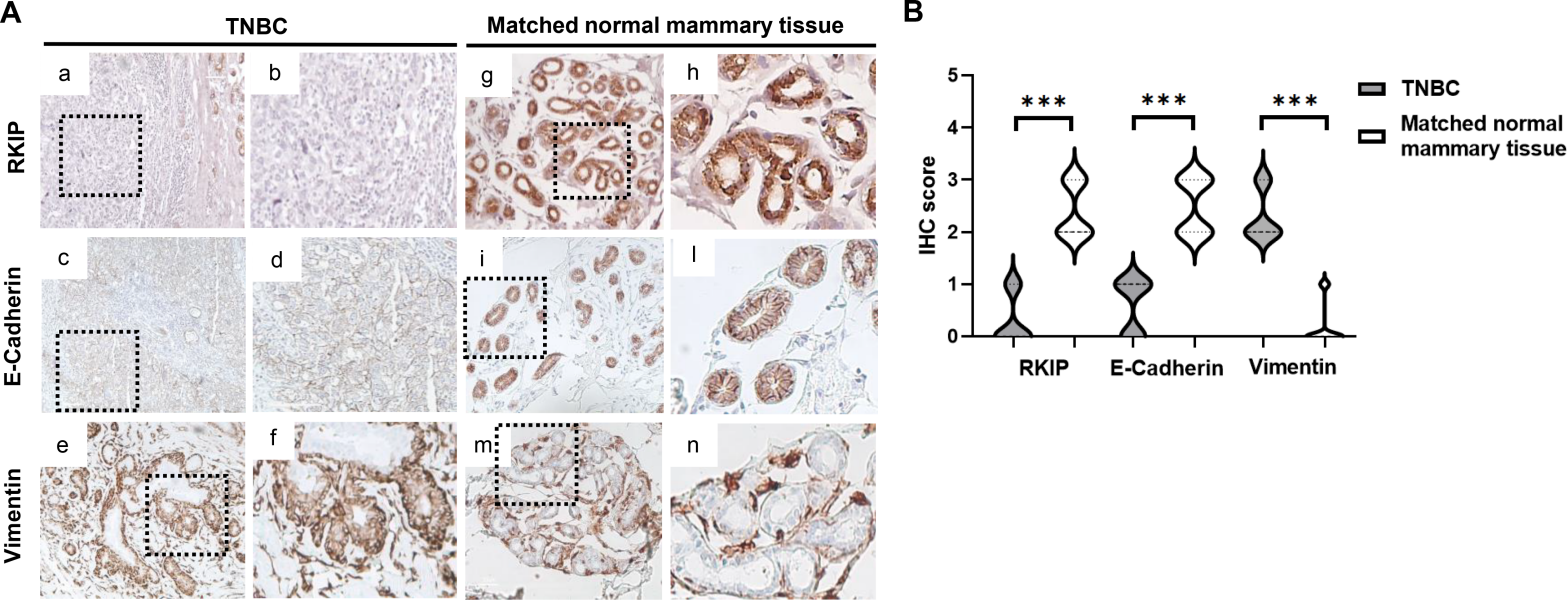
**(A)** Representative IHC images of RKIP, E-Cadherin, and Vimentin protein expression in TNBC (a–f) and matched normal mammary (g–n) tissue samples. Magnification, ×20 and ×40. **(B)** Violin plots of RKIP, E-Cadherin, and Vimentin IHC score in TNBC and matched normal mammary tissue samples. ***p-value < 0.001. IHC, immunohistochemistry; RKIP, Raf kinase inhibitor protein; TNBC, triple-negative breast cancer.

At IHC analysis, TNBC tissue samples were negative for RKIP expression (score ≤ 1) in all samples analyzed, concomitant with a decrease in E-Cadherin membrane positivity and an increase in Vimentin expression in the cytoplasm of tumor cells (**Figure 1A, a–f**). Conversely, matched normal mammary tissues showed a strong cytoplasmic positivity for RKIP expression (score ≥ 2), associated with a high membrane expression of the epithelial marker E-Cadherin, whereas Vimentin staining was confined only to stromal cells (**Figure 1A, g–n**). Overall, these findings showed that RKIP and E-Cadherin expression were significantly downregulated, while Vimentin expression was upregulated, in TNBC compared to matched normal mammary tissues (**Figure 1B**).

### Gabexate mesylate upregulates RKIP expression and inhibits p44/42 MAPK and NF-κB signaling in MDA-MB-231 cells

For *in vitro* studies, the MDA-MB-231 cell line, a well-recognized TNBC cell model enriched in EMT traits and with highly aggressive features (20, 21), was selected. Preliminary experiments on MDA-MB-231 cell viability were carried out in order to prevent cytotoxic effects secondary to GM administration. No effects on cell viability were observed with the dose of 50 μg/mL over 72 hrs (**Supplementary Figure 1**); accordingly, this schedule of treatment was adopted in subsequent experiments.

**Figure 2:**
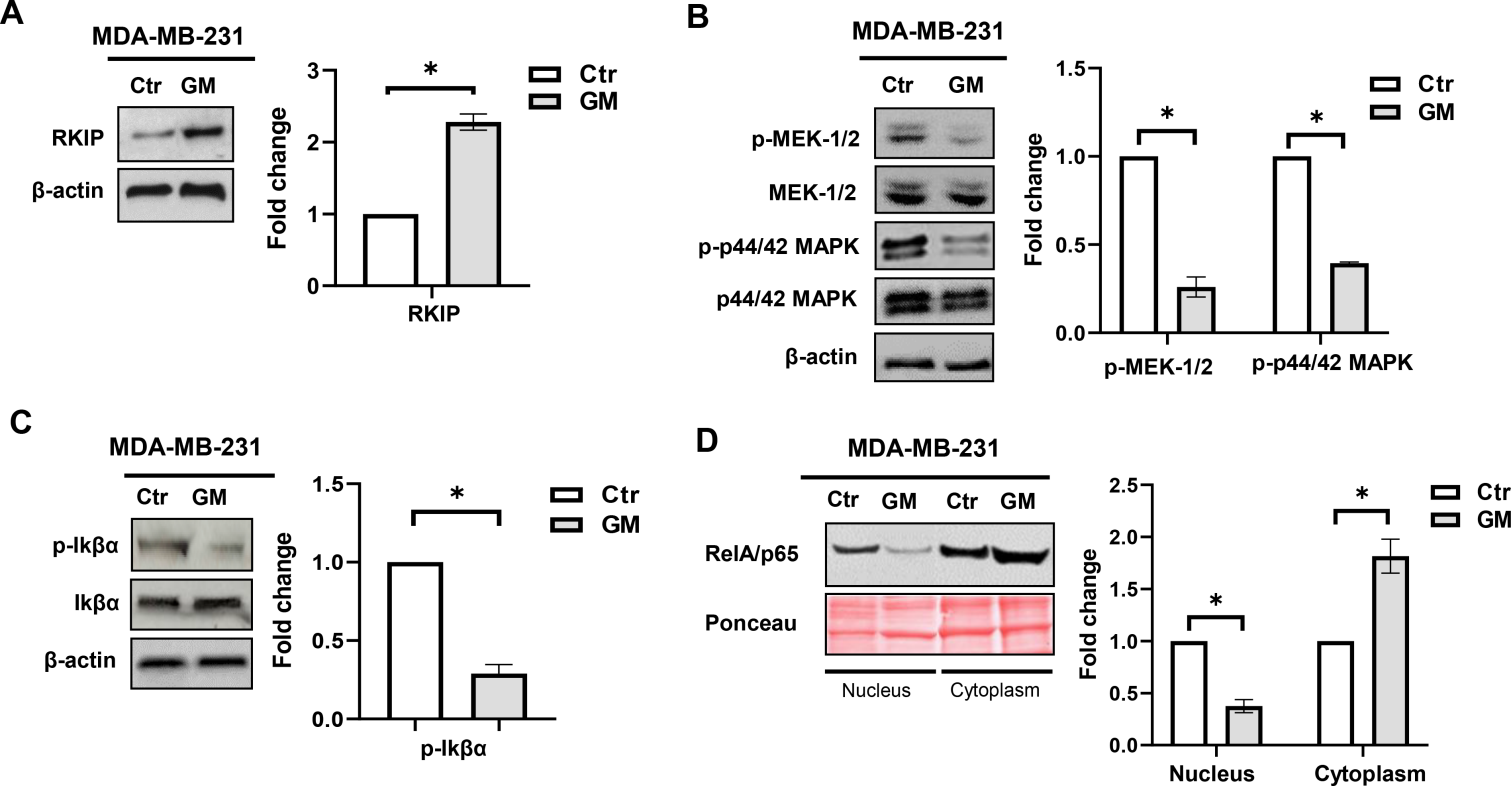
Western blotting analysis of RKIP **(A)**, phosphorylated MEK-1/2 and p44–42 MAPK **(B)**, and phosphorylated Ikβα **(C)** protein expression in MDA-MB-231 cells treated with GM 50 µg/mL for 72 hrs. Histograms show RKIP, phosphorylated MEK-1/2, p44–42 MAPK, and Ikβα fold change in treated cells relative to controls, which were assigned a value of 1. Each sample was run in duplicate. β-Actin, total MEK-1/2, p44–42 MAPK, and Ikβα protein were used for normalization, as appropriate. Representation of three separate experiments with similar findings. **(D)** Western blotting analysis of RelA/p65 protein expression in the nuclear and cytoplasmic fractions of MDA-MB-231 cells treated with GM 50 µg/mL for 72 hrs. Histograms show RelA/p65 fold change in treated cells relative to controls, which were assigned a value of 1. Each sample was run in duplicate, and Ponceau red was used for equal protein loading and normalization. Representation of three separate experiments with similar findings. *p-value <; 0.05. Ctr, control; GM, gabexate mesylate; RKIP, Raf kinase inhibitor protein; MAPK, mitogen-activated protein kinase.

We first investigated whether treatment with GM could upregulate RKIP expression in TNBC cells, similarly to what we observed in pancreatic cancer cells (14). We found that administration for 72 hrs of GM induced a significant increase in RKIP expression in MDA-MB-231 cells (**Figure 2A**). Then, as RKIP is a physiological endogenous inhibitor of the p44/42 MAPK signaling, we investigated the effect of GM treatment on this pathway. As shown in **Figure 2B**, a significant decrease in phosphorylated MEK-1/2 and p44/42 MAPK proteins was observed in this cell line following GM treatment, confirming the inhibition of MAPK signaling by this drug.

Due to the role of RKIP also in the regulation of NF-κB signaling (8), we further investigated the effect of GM on this oncogenic pathway. It is known that, in the absence of stimuli, IkBα exerts an inhibitory effect on NF-κB signaling through the formation of a complex with RelA/p65 protein in the cytosol. When the canonical NF-κB pathway is activated, IkBα phosphorylation occurs, leading to its proteasome-dependent degradation, and the RelA-p50 dimer may enter the nucleus, acting as a transcription factor. As reported in **Figure 2C**, GM administration decreased the expression of phosphorylated IkBα protein; moreover, subcellular fractionation of treated cells showed an increased level of RelA/p65 cytoplasmic protein expression, concomitant with a decrease in the nuclear fraction (**Figure 2D**).

Overall, these findings show that GM upregulates RKIP expression, dampening both p44/42 MAPK and NF-κB pathways in MDA-MB-231 cells.

### Gabexate mesylate reverses EMT phenotype in MDA-MB-231 cells

**Figure 3:**
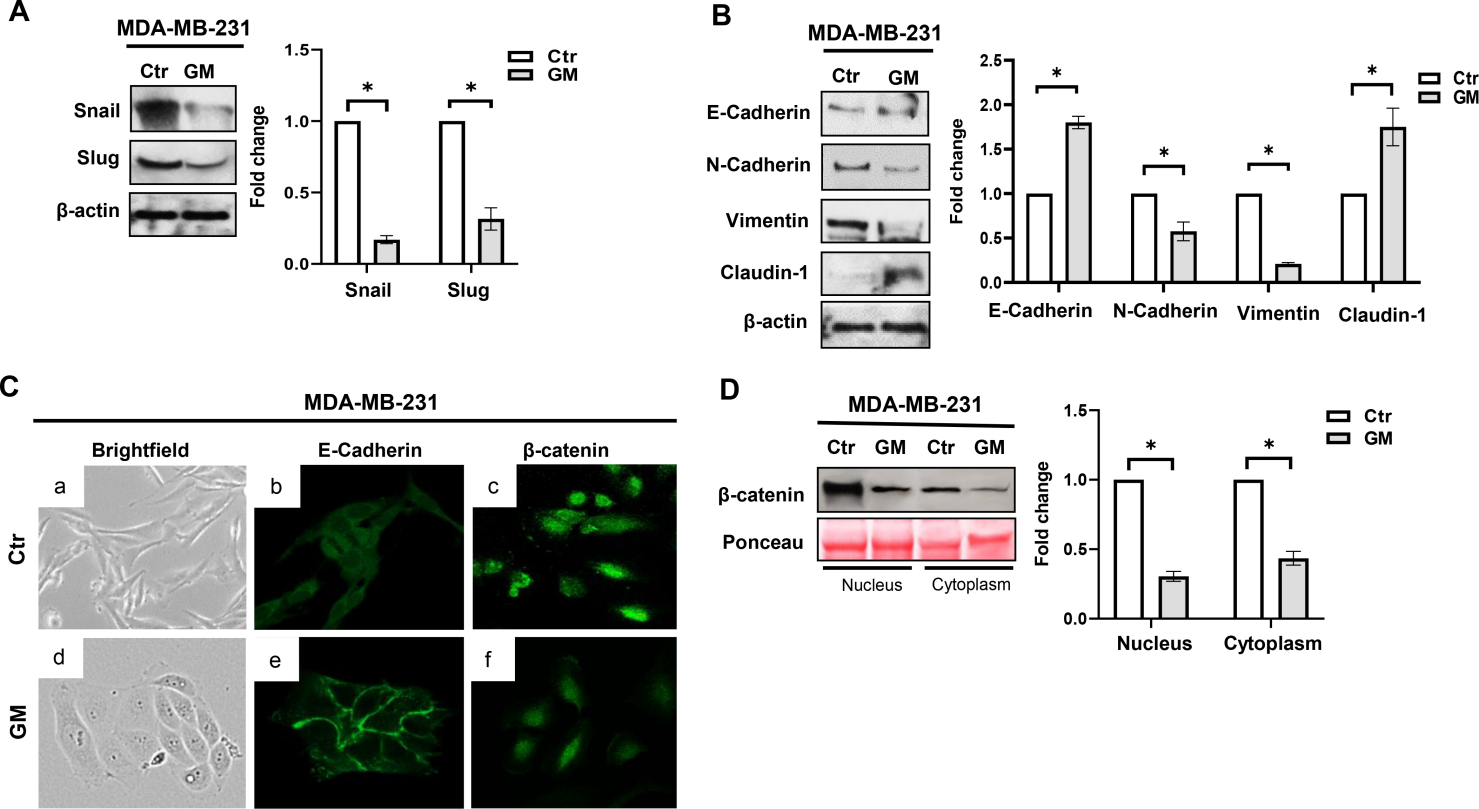
**(A, B)** Western blotting analysis of Snail, Slug, E-Cadherin, N-Cadherin, Vimentin, and Claudin-1 protein expression in MDA-MB-231 cells treated with GM 50 µg/mL for 72 hrs. Histograms show Snail, Slug, E-Cadherin, N-Cadherin, Vimentin, and Claudin-1 fold change in treated cells relative to controls, which were assigned a value of 1. Each sample was run in duplicate, and β-actin was used for equal protein loading and normalization. Representation of three separate experiments with similar findings. **(C)** Phase-contrast microscopy images (a, d) and confocal microscopy analysis of E-Cadherin (b, e) and β-catenin (c, f) cellular localization in MDA-MB-231 cells after 72 hrs of treatment with Vehicle (Ctr) or GM 50 µg/mL. Images are representative of three independent experiments with similar findings. Magnification, ×40. **(D)** Western blotting analysis of β-catenin protein expression in the nuclear and cytoplasmic fractions of MDA-MB-231 cells treated with GM 50 µg/mL for 72 hrs. Histograms show β-catenin fold change in treated cells relative to controls, which were assigned a value of 1. Each sample was run in duplicate, and Ponceau red was used for equal protein loading and normalization. Representation of three separate experiments with similar findings. * p-value <; 0.05. Ctr, control; GM, gabexate mesylate.

The activation of p44/42 MAPK and NF-κB pathways has been reported to induce the expression of some EMT transcription factors, playing a critical role in the transcriptional repression of epithelial cell characteristics and the promotion of mesenchymal traits (22, 23). Accordingly, we investigated whether the inhibition of p44/42 MAPK and NF-κB pathways by GM was associated with a decreased expression of the EMT transcription factors Snail and Slug. As reported in **Figure 3A**, treatment with this drug for 72 hrs significantly reduced the levels of both proteins. This decrease was concomitant with an increased expression of the epithelial markers E-Cadherin and Claudin-1, and a decrease in the mesenchymal markers N-Cadherin and Vimentin (**Figure 3B**). At the morphological level, treatment with GM induced a switch from an elongated/fibroblastic to a more epithelial cell morphology of MDA-MB-231 cells (**Figure 3C, a, d**), suggesting the promotion of mesenchymal–epithelial transition (MET). Because during EMT the expression of the cell-adhesion molecule E-Cadherin decreases, weakening cell–cell junctions and leading to cytoplasmic accumulation and nuclear translocation of β-catenin (24), we also investigated the effect of GM treatment on E-Cadherin and β-catenin cellular localization. By confocal analysis, we observed E-Cadherin membrane localization in MDA-MB-231-treated cells (**Figure 3C, b, e**), along with a reduction of both cytoplasmic and nuclear β-catenin staining (**Figure 3C, c, f**), thus confirming that GM treatment can increase cell–cell junctions and promote the MET phenotype in these cells.

### Gabexate mesylate affects MDA-MB-231 cell motility and invasiveness

**Figure 4:**
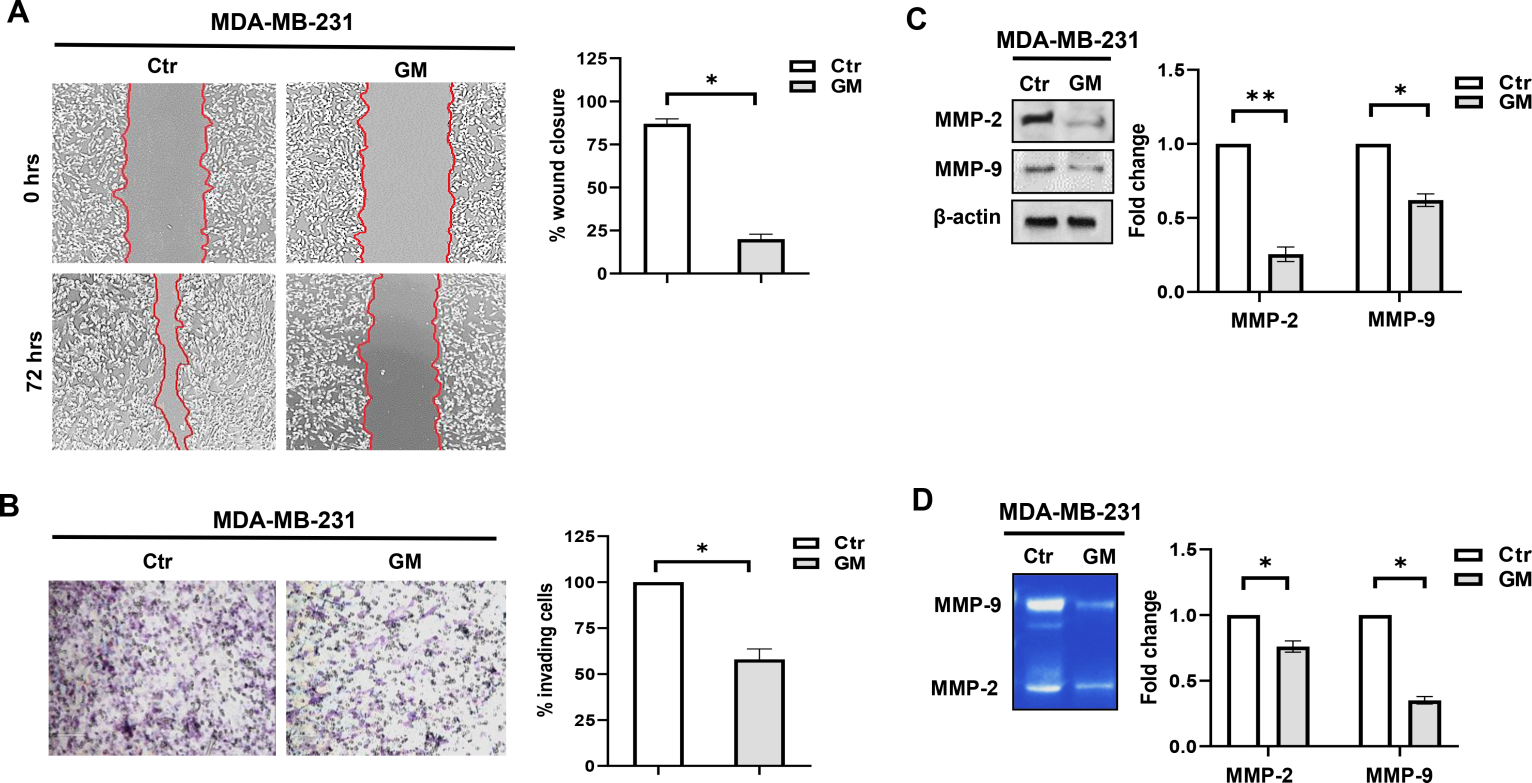
**(A)** Wound-healing scratch assay in MDA-MD-231 cells at baseline (T0) and after 72 hrs of treatment with Vehicle (Ctr) or GM 50 µg/mL. Images are representative of three independent experiments with similar findings. Magnification, ×10. Histograms show densitometric analysis of wound closure percentage in Ctr and treated cells. **(B)** Evaluation of invasive potential in MDA-MD-231 cells after 72 hrs of treatment with Vehicle (Ctr) or GM 50 µg/mL. Images are representative of three independent experiments with similar findings. Magnification, ×10. Histograms show the percentage of invading cells in Ctr and treated cells. **(C)** Western blotting analysis of MMP-2 and MMP-9 protein expression in MDA-MD-231 cells treated for 72 hrs with GM 50 µg/mL. Histograms show MMP-2 and MMP-9 fold change in treated cells relative to controls, which were assigned a value of 1. Each sample was run in duplicate, and β-actin was used for equal protein loading and normalization. Representation of three separate experiments with similar findings. **(D)** Evaluation of MMP-2 and MMP-9 protein activities in the conditioned medium of MDA-MD-231 cells treated for 72 hrs with GM 50 µg/mL. Histograms show MMP-2 and MMP-9 gelatinolytic activity fold change in treated cells relative to controls, which were assigned a value of 1. Each sample was run in duplicate. Representation of three separate experiments with similar findings. *p-value <; 0.05; **p-value <; 0.01. Ctr, control; GM, gabexate mesylate.

Finally, we investigated whether GM-induced switch from the EMT to MET phenotype was also mirrored by a decrease in MDA-MB-231 cell motility and invasive potential. Scratch assay showed that 72 hrs of treatment with GM significantly decreased MDA-MB-231 cell migration, compared to control cells, which tended to close the wound gap (**Figure 4A**); similarly, GM-treated cells displayed a reduced invasive behavior compared to controls (**Figure 4B**). At the molecular level, we observed a significant reduction of both MMP-2 and MMP-9 protein expression and activity in GM-treated cells (**Figures 4C, D**). Overall, these findings indicate that GM is able to reverse the EMT phenotype and significantly reduce the migration and invasive potential of TNBC cells.

## Discussion

Over the years, it has become increasingly clear that TNBCs are a group of heterogeneous malignancies at the genomic, transcriptomic, and histopathological levels (25). Even though many efforts have been made to identify putative molecular subgroups with prognostic or therapeutic relevance, their current clinical advantages remain quite ancillary (26). Not surprisingly, the identification of single actionable targets and, hence, biomarker-driven therapy, is still an unmet clinical need. The use of targeted therapies, such as Poly-ADP ribose polymerase (PARP), tyrosine-kinase inhibitors, immunotherapy, and Chimeric Antigen Receptor T-cell therapy (CAR-T) cells, rarely represents a valuable therapeutic option (27, 28). Rather, few patients could currently take advantage of some tailored approaches, including platinum agents or PARP inhibitors in patients harboring BRCA1/2 germline mutation, anti-TROP2-conjugated cytotoxic drug (sacituzumab govitecan) in advanced-stage TNBC, and, finally, immunotherapy in PD-L1-expressing tumors (29–35). It is conceivable that the continuous progress in either RNA- or DNA-based Next-generation sequencing (NGS) technologies may extend the number of patients eligible for targeted therapeutic strategies. Nevertheless, several obstacles in unraveling the tumoral heterogeneity conundrum still make standard chemotherapy the cornerstone in the treatment of TNBC (36–38). In this scenario, TNBC patients still have a poor prognosis, basically attributable to rapid disease progression and early metastases involving critical organs.

**Figure 5:**
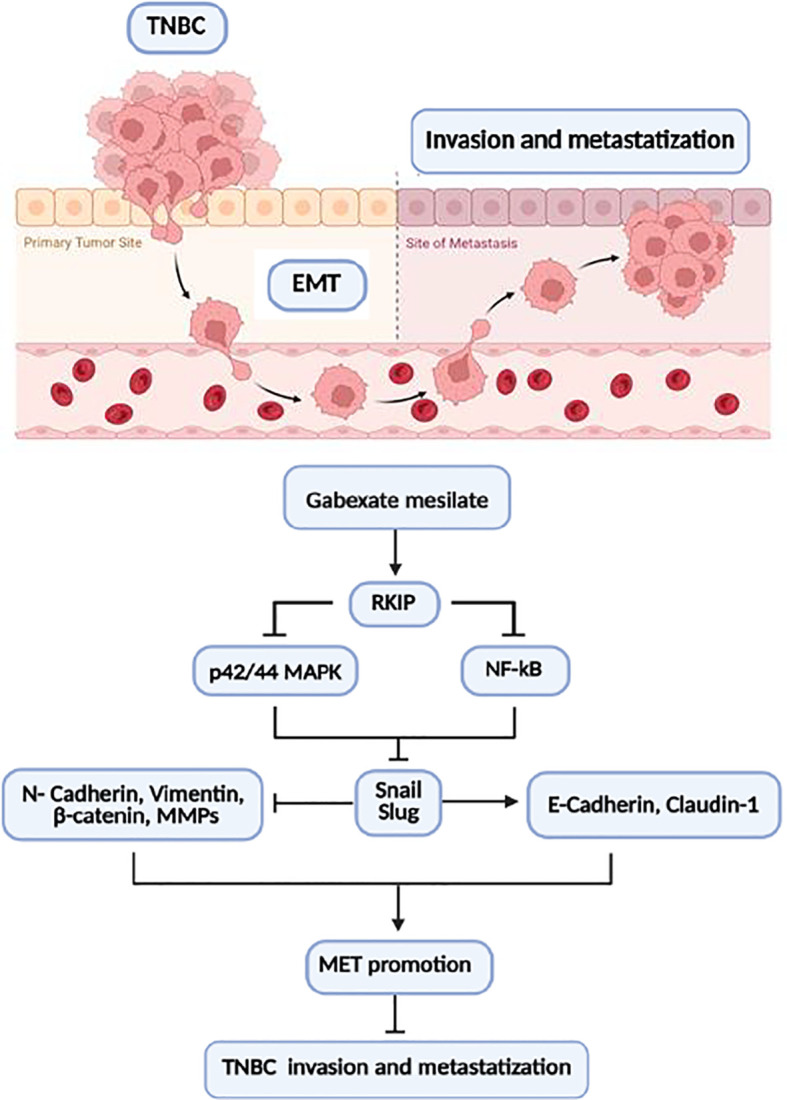
Schematic representation of EMT inhibition following GM treatment in MDA-MD-231 TNBC cells. Inhibition of RKIP expression by GM results in inhibition of p42/44 MAPK and NF-κB signaling, which in turn decreases the expression of the EMT transcription factors Snail and Slug. Parallelly, the decrease in Snail and Slug upregulates E-Cadherin and Claudin-1 protein expression and downregulates N-Cadherin, Vimentin, β-catenin, and MMP protein expression, promoting MET phenotype. TNBC, triple-negative breast cancer; EMT, epithelial–mesenchymal transition; MET, mesenchymal–epithelial transition.

Emerging studies have suggested that TNBC metastatization is related, at least in part, to cancer cell EMT phenotype. Accordingly, the use of effective drugs targeting EMT has been suggested as an attractive therapeutic approach to improve the clinical outcome of TNBC patients (39, 40). In terms of therapeutic implications, the wide span of signaling pathways implicated in the orchestration of the EMT process offers various potential actionable molecular targets. Although several classes of drugs have been put in place over the years, almost all failed the benchmark of clinical trial (41–44). Thus, a pharmacological approach directed to contrast EMT-driven tumor aggressiveness and metastatic potential in TNBCs is still lacking. To date, only eribulin, a non-taxane spindle poison approved for metastatic or locally advanced TNBC, has been demonstrated to be effective in contrasting EMT-driven phenotype (45, 46). Among the molecular alterations driving TNBC metastasis, dysregulation of the RKIP pathway has emerged in several studies (9–13). The identification of drugs able to restore RKIP expression is gaining increasing scientific attention as a potential therapeutic strategy in these patients.In the present study, we reported that the expression of the metastasis suppressor gene RKIP is lost in TNBC tissue samples and is associated with the upregulation of the mesenchymal marker Vimentin and the downregulation of the epithelial marker E-Cadherin. These findings are in line with the well-known high aggressiveness and metastatic potential of this BC subtype. Interestingly, we demonstrated that treatment with the protease inhibitor GM, in a dose clinically achievable in human blood, was able to increase RKIP expression and revert the EMT phenotype in a well-established *in vitro* TNBC model. This phenotypic change was concomitant with the upregulation of the epithelial markers E-Cadherin and Claudin-1, and the downregulation of the mesenchymal markers N-Cadherin, Vimentin, and nuclear β-catenin. Furthermore, GM significantly decreased *in vitro* TNBC cell motility and invasiveness, along with MMP-2 and MMP-9 protein expression and activity, which represent negative prognostic factors in TNBC (47–49). At the mechanistic level, RKIP upregulation inhibited p42/44 MAPK and NF-κB signaling that, in turn, downregulated the expression of the EMT transcription factors Snail and Slug (**Figure 5**).

Despite these preliminary findings needing to be confirmed in more representative *ex vivo* (such as patient-derived primary cells or patient-derived organoids) and *in vivo* TNBC models, they provide evidence that the EMT process can be pharmacologically reverted by the protease inhibitor GM in a highly metastatic TNBC *in vitro* model, deserving further investigation in future studies.

## Data Availability

The raw data supporting the conclusions of this article are available at the following link: https://zenodo.org/records/18456746.

## Supplementary material

Supplementary Figure 1 Cell viability assay in MDA-MB-231 cells treated for 24, 48 and 72 hrs with vehicle (Ctr) or GM 50µg/mL. O.D. 570nm is proportional to the number of viable cells in the sample. Each sample was run in triplicate. Data are presented as mean OD ± SEM of three independent experiments. OD, optical density; Ctr, control; GM, gabexate mesylate.
