# Supplementary figures and images for: The protease inhibitor gabexate mesylate targets Raf kinase inhibitor protein and reverses epithelial–mesenchymal transition in triple-negative breast cancer cells
Source: Front Oncol. 2026 Mar 2;16:1713273. doi: 10.3389/fonc.2026.1713273 (PMC12989336; doi:10.3389/fonc.2026.1713273)

## Slide 1
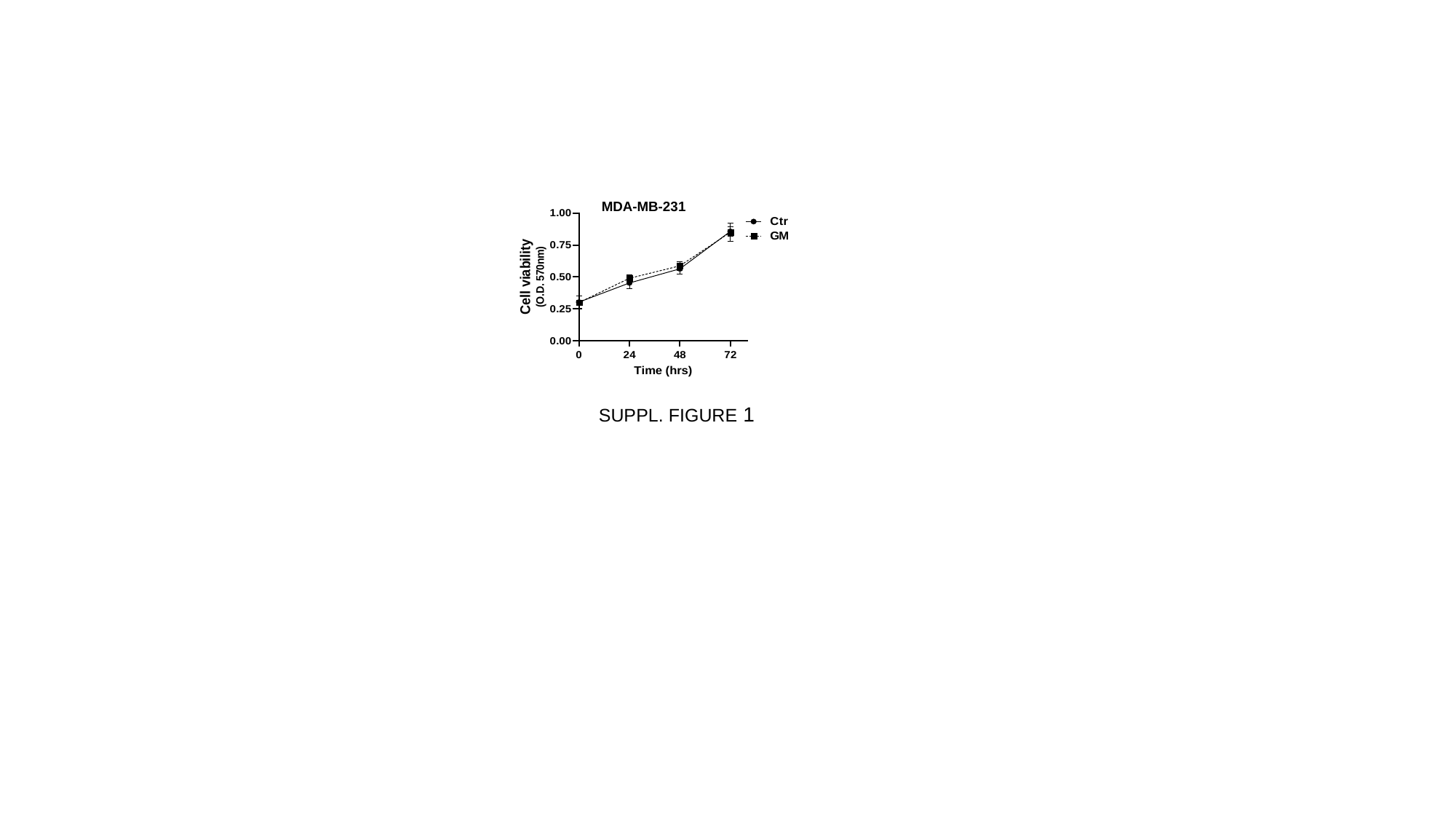

MDA-MB-231
SUPPL. FIGURE 1

Supplement: Supplementary Figure 1 — Cell viability assay in MDA-MB-231 cells treated for 24, 48 and 72 hrs with vehicle (Ctr) or GM 50µg/mL. O.D. 570nm is proportional to the number of viable cells in the sample. Each sample was run in triplicate. Data are presented as mean OD ± SEM of three independent experiments. OD, optical density; Ctr, control; GM, gabexate mesylate. [file SupplementaryFile1.pptx]
